# Hypoglycemia-Induced Irreversible Brain Injury: A Case Report

**DOI:** 10.7759/cureus.86912

**Published:** 2025-06-28

**Authors:** Aakash Bisht, Saliha Erdem, Richa Gupta, Hassan Makki

**Affiliations:** 1 Internal Medicine, Detroit Medical Center, Wayne State University, Detroit, USA; 2 Pediatrics, Government Medical College, Patiala, Patiala, IND; 3 Pulmonary, Critical Care and Sleep Medicine, Wayne State University School of Medicine, Detroit, USA

**Keywords:** anoxic brain injury, hypoglycemia-induced brain injury, irreversible brain injury, low blood glucose, persistent hypoglycemia

## Abstract

Hypoglycemia-induced brain injury, though less common than hyperglycemia, can lead to significant neurological sequelae and mortality. We present a case of a 76-year-old female with a history of poorly controlled diabetes who presented with altered mental status and unresponsiveness. Despite the correction of hypoglycemia and supportive medical management, the patient's neurological status did not improve. Neurological examination revealed myoclonus and severe encephalopathy, prompting further investigation. Imaging studies, including contrast-enhanced CT and MRI of the head, demonstrated findings suggestive of metabolic brain injury, consistent with hypoglycemia-induced encephalopathy. Despite treatment, the patient's condition did not improve, and a decision was made to transition towards comfort-focused care with palliative extubation. This case underscores the challenges in managing hypoglycemia-induced encephalopathy and highlights the importance of timely recognition and intervention in preventing irreversible neurological damage.

## Introduction

Hypoglycemia-induced brain injury, also known as hypoglycemic encephalopathy, poses a significant threat to life and neurological function, particularly in cases of severe, prolonged hypoglycemia with serum glucose levels less than 20 milligrams per deciliter (mg/dL). While commonly associated with diabetes mellitus, hypoglycemia may also affect non-diabetic individuals [1]. Prevalence of at least a single episode of severe hypoglycemia ranged from 6 in 100 patient years in patients with type 1 diabetes mellitus (T1DM) and 0.7 to 12 in 100 patient years in patients with T2DM [2,3]. Non-diabetic patients make up less than 0.1% of total admissions presenting with severe hypoglycemia [1]. The severity and duration of low blood glucose levels determine the extent of neuronal damage, which can range from reversible to permanent [4]. Symptoms typically manifest initially as sweating, palpitations, confusion, dizziness, and gait instability, which may progress to drowsiness, lethargy, and coma if left untreated. Management strategies vary based on the severity of hypoglycemia and may include oral glucose paste, glucagon injections, and intravenous administration of concentrated dextrose fluids [5].

To illustrate the devastating consequences of severe hypoglycemia, we present a case of a 76-year-old female with a recent history of hyperglycemia, who presented with altered mental status and was found to be severely hypoglycemic. Despite supportive medical management, including correction of hypoglycemia, the patient's condition did not improve, leading to a decision to transition towards comfort-focused care with palliative extubation. This case underscores the importance of timely recognition and intervention in hypoglycemic emergencies to prevent irreversible neurological sequelae and improve patient outcomes.

## Case presentation

A 76-year-old cachectic female was brought to the emergency department (ED) via emergency medical services due to altered mental status. As per history obtained from her son, the patient's last known well time was around 23:30 the previous night and was subsequently found unresponsive the next afternoon at approximately 14:00. The son further elaborated that the patient was recently discharged from a skilled nursing facility after a month-long stay. Prior to that, she had been hospitalized for more than a week due to severe hyperglycemia, during which her presenting blood sugar was found to be 1760 mg/dL. Her baseline state included relative independence with moderate assistance for daily living activities. Her past medical history was significant for hypertension, hyperlipidemia, T2DM, dysphagia, and adult failure to thrive. She had no known previous drug allergies. Home medications included amlodipine 5 milligrams (mg) once daily (QD), lisinopril 40 mg QD, metoprolol succinate 100 mg QD, atorvastatin 80 mg once daily at bedtime (QHS), rapid-acting insulin lispro 10 units three times daily before meals (TIDAC), and long-acting insulin glargine 30 mg QHS. Compliance with medications was unknown.

Vital signs on admission were notable for hypertension and tachycardia with blood pressure of 140/71 millimeters of mercury (mmHg) and heart rate of 117 beats per minute (bpm). She was saturating well on room air at 100% with a temperature of 36.7 degrees Celsius (°C). Initial examination revealed the patient to be unresponsive with a Glasgow Coma Scale (GCS) score of 3, exhibiting facial twitches, right-sided eye deviation, and frequent twitching of the right upper extremity. Finger stick glucose was found to be low at 16 mg/dL, and subsequently, the patient was given two 25 milliliters (mL) ampoules of 50% dextrose injection, leading to an increase in fingerstick glucose level to 183 mg/dL. Despite this correction, the patient's mentation did not improve, prompting intubation for airway protection. The patient was also given lorazepam 4 mg and loaded with levetiracetam 3 grams in two divided doses for possible seizure-like activity, and the patient was transferred to the Medical Intensive Care Unit (MICU) for further treatment.

Laboratory findings showed several abnormal findings (Table 1). Severe hypoglycemia was noted with a blood glucose level of 16 mg/dL, mild hyponatremia with serum sodium level of 133 millimoles per liter (mmol/L), and an elevated creatine phosphokinase level of 523 units per liter (u/L). Complete blood count (CBC) showed normocytic anemia with a hemoglobin level of 9.7 grams per deciliter (g/dL) and mean corpuscular volume (MCV) of 91.7 femtoliters (fL). High sensitivity troponin I levels were found to be elevated at 36 nanograms per liter (ng/L) but declined after four hours to a level of 24 ng/L. Other lab findings on presentation included serum potassium level of 4.4 mmol/L, serum chloride level of 102 mmol/L, serum bicarbonate level of 24 mmol/L, serum calcium level of 9.3 mg/dL, serum magnesium level of 2.4 mg/dL, serum phosphorus level of 4.4 mg/dL, serum blood urea nitrogen level of 21 mg/dL and serum creatinine level of 0.97 mg/dL. Serum lactic acid was found to be 1.8 mmol/L with ammonia level of 34 micromoles per L (µmol/L). White blood cells (WBC) and platelet count were within normal limits, with levels of 9300 cells per microliter (cells/µL) and 204,000 cells/µL, respectively. Liver function tests were reported as alanine aminotransferase (ALT) level of 33 u/L, aspartate aminotransferase (AST) level of 30 u/L, alkaline phosphatase (ALP) level of 61 u/L, serum total bilirubin level of 0.47 mg/dL with total protein level of 5.7 g/dL and serum albumin level of 3.4 g/dL. Urinalysis showed a potential of hydrogen (pH) level of 6.0, urine glucose 2+, and urine protein 2+ but was otherwise insignificant. Urinary drug screen was negative for amphetamines, barbiturates, benzodiazepines, cannabinoids, cocaine metabolites, methadone, and opiates. Sulfonylurea and alcohol screen were also performed, and the results were reported as negative.

**Table 1:** Laboratory findings with reference values "L" indicates patient values below the reference range, "H" indicates patient values above the reference range. mg/dL: milligrams per deciliter; mmol/L: millimoles per liter; µmol/L: micromoles per liter; u/L: units per liter; ng/L: nanograms per liter; g/dL: grams per deciliter; fL: femtoliters; cells/µL: cells per microliter; cells/hpf: cells per high power field; MCV: mean corpuscular volume; WBC: white blood cells; ALT: alanine aminotransferase; AST: aspartate aminotransferase; ALP: alkaline phosphatase; pH: potential of hydrogen; RBC: red blood cells

Laboratory	Patient Values		Reference Values
Serum chemistry			
Glucose (mg/dL)	16 (L) (initial)		75-105
	183 (H) (repeat)		
Sodium (mmol/L)	133 (L)		135-145
Potassium (mmol/L)	4.4		3.5-5.1
Chloride (mmol/L)	102		98-107
Bicarbonate (mmol/L)	24		21-31
Calcium (mg/dL)	9.3		8.6-10.8
Magnesium (mg/dL)	2.4		1.6-3.0
Phosphorus (mg/dL)	4.4		2.5-4.5
Blood urea nitrogen (mg/dL)	21		7-25
Creatinine (mg/dL)	0.97		0.60-1.20
Lactic acid (mmol/L)	1.8		0.4-2.0
Ammonia (µmol/L)	34		16-53
Creatinine phosphokinase (µ/L)	523 (H)		30-223
Troponin (ng/L)	36 (H) (initial)		3-17
	24 (H) (repeat)		
Hematology			
Hemoglobin (g/dL)	9.7 (L)		11.5-15.1
MCV (fL)	91.7		82-97
WBC (cells/µL)	9300		3500-10,600
Platelet (cells/µL)	2,04,000		150,000-450,000
Liver function test			
ALT (u/L)	33		7-52
AST (u/L)	30		13-39
ALP (u/L)	61		50-142
Total bilirubin (mg/dL)	0.47		<1.50
Total protein (g/dL)	5.7 (L)		6.4-8.9
Albumin (g/dL)	3.4 (L)		3.5-5.7
Urinalysis			
Urine pH	6		5.0-8.5
Urine glucose	2+		Negative
Urine protein	2+		Negative
Urine leukocyte esterase	Negative		Negative
Urine nitrite	Negative		Negative
Urine bacteria	Negative		Negative
Urine RBC (cells/hpf)	<2		<2
Urine WBC (cells/hpf)	<2		<5
Toxicology			
Urine amphetamines	Negative		Negative
Urine barbiturates	Negative		Negative
Urine benzodiazepines	Negative		Negative
Urine cannabinoids	Negative		Negative
Urine cocaine metabolite	Negative		Negative
Urine methadone	Negative		Negative
Urine opiates	Negative		Negative
Sulfonylurea	Negative		Negative
Alcohol (mg/dL)	<10		<80

**Figure 1:**
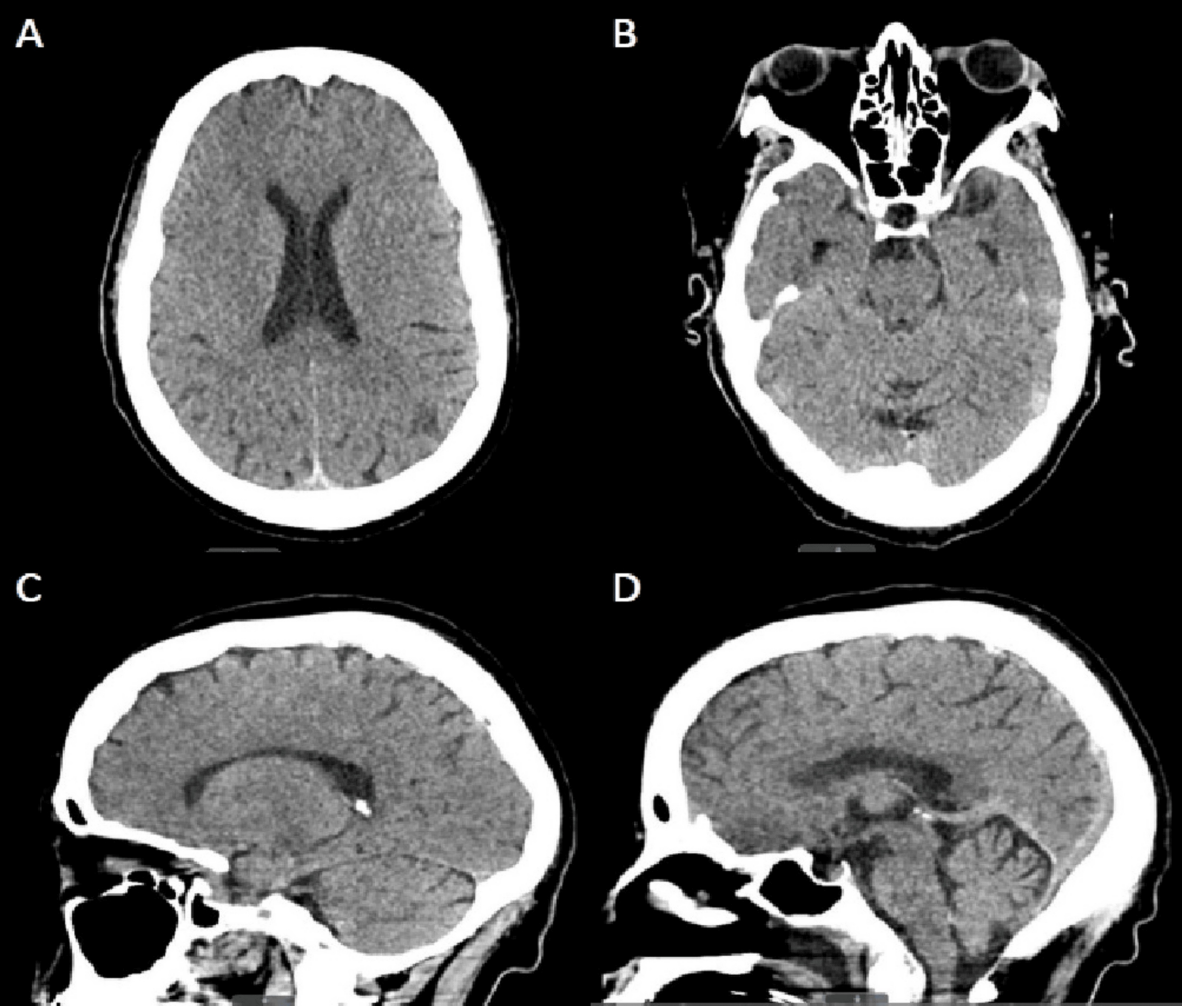
Non-contrast CT of the head showing no acute intracranial process. (A) Axial image at the level of the lateral ventricles; (B) Axial image at the level of the pons; (C) Sagittal image at the level of the thalamus; (D) Sagittal image at the level of the central sulcus.

**Figure 2:**
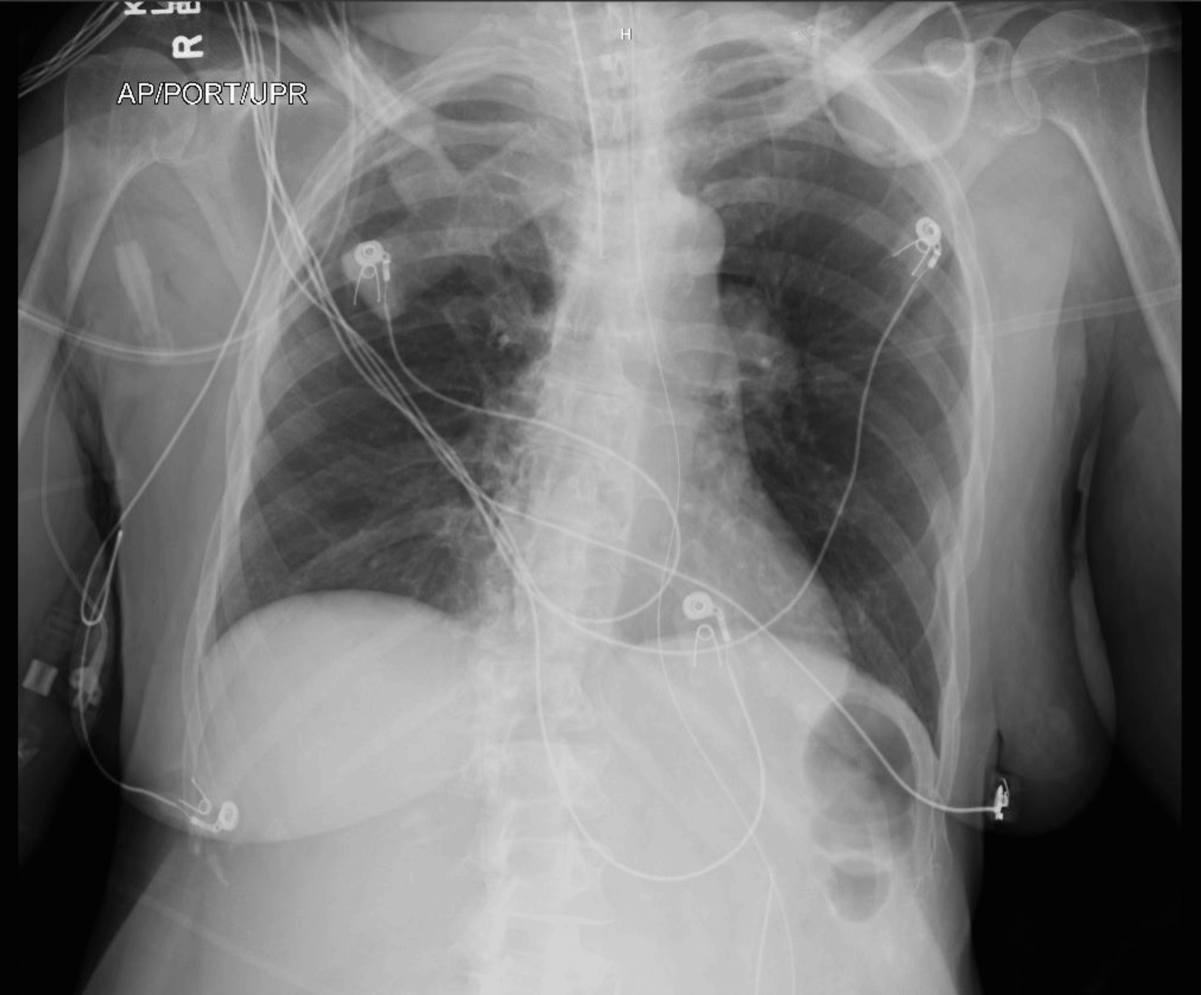
Portable upright anteroposterior chest X-ray showing no cardiopulmonary abnormalities.

Computed tomography (CT) of the head without intravenous contrast on presentation did not reveal any evidence of acute intracranial hemorrhage or other intracranial abnormalities (Figure 1). Chest X-ray did not reveal any cardiopulmonary abnormalities (Figure 2). Empirical antibiotic therapy with vancomycin and cefepime was initiated due to concerns for possible infection. However, the patient's infectious workup remained negative throughout her hospitalization, including chest X-ray, urinalysis, blood, and respiratory cultures. Therefore, empiric antibiotics were discontinued on day 3 of hospitalization.

**Figure 3:**
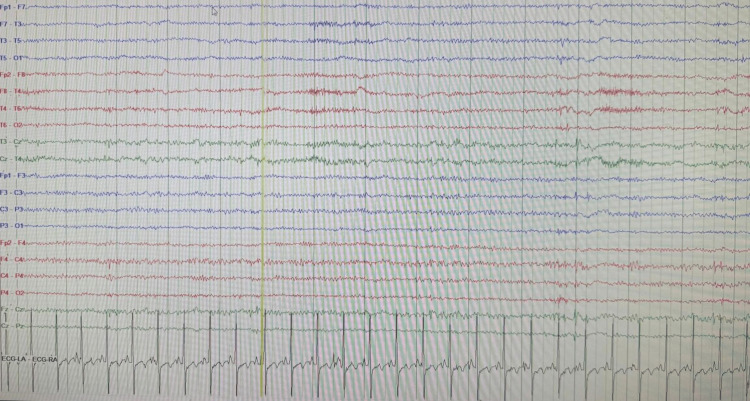
Electroencephalogram (EEG) study showing lack of normal background activity.

**Figure 4:**
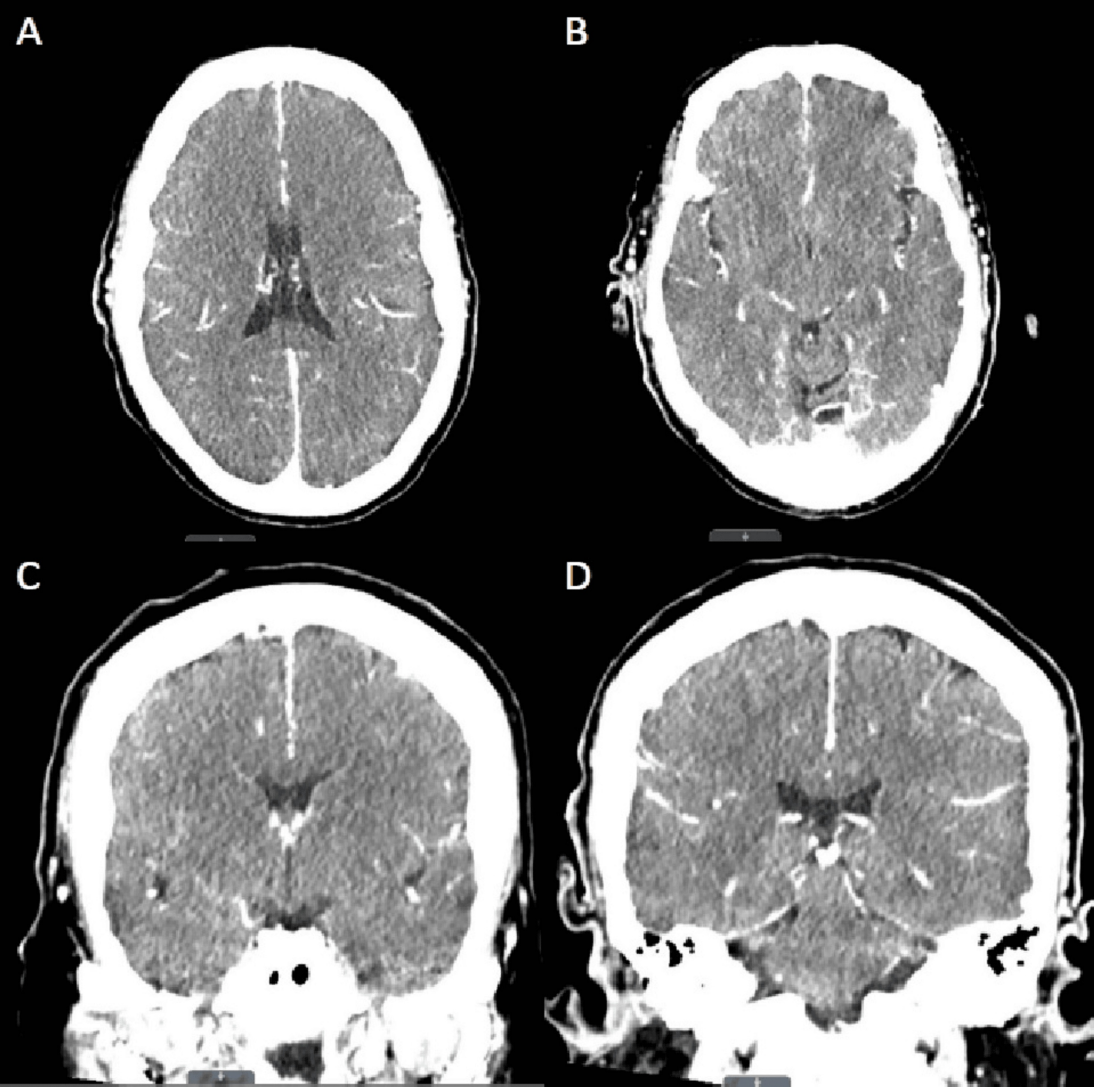
Contrast-enhanced CT of the head showing mild cerebral edema. (A) Axial image at the level of the lateral ventricles; (B) Axial image at the level of the aqueduct of Sylvius; (C) Coronal image at the level of the optic tract; (D) Coronal image at the level of the superior colliculus.

**Figure 5:**
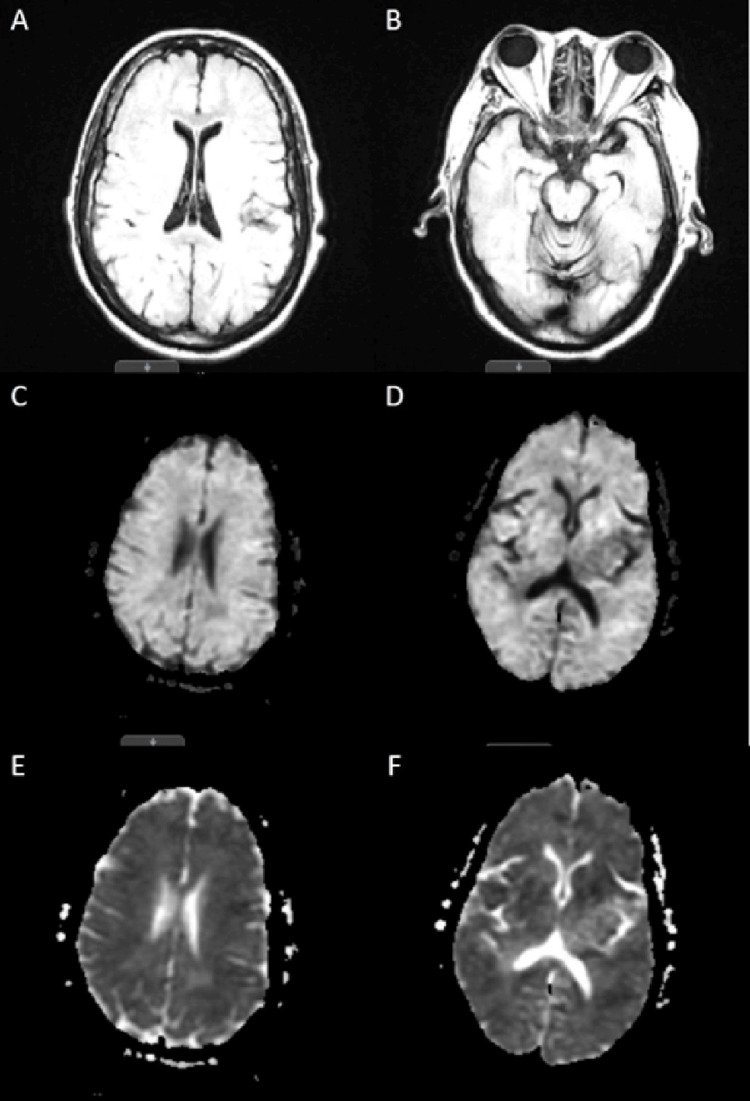
Non-contrast MRI of the head showing diffuse diffusion restriction, partial sulcal effacement, and mild diffuse cerebral edema. (A) Axial T2-weighted fluid-attenuated inversion recovery (FLAIR) image at the level of the frontal horn of the lateral ventricle. (B) Axial T2-weighted FLAIR image at the level of the midbrain. (C) Axial diffusion-weighted image (b1000) at the level of the lateral ventricles and inferior sagittal sinus. (D) Axial diffusion-weighted image (b1000) at the level of the atrium of the lateral ventricle. (E) Axial apparent diffusion coefficient (ADC) image at the level of the lateral ventricles and inferior sagittal sinus. (F) Axial ADC image at the level of the atrium of the lateral ventricle.

During her stay in the MICU, the patient exhibited frequent twitches and abnormal jerking movements of her right upper extremity. Multiple continuous electroencephalogram (EEG) studies were conducted, revealing a markedly abnormal pattern characterized by a lack of normal background activity and low voltage fast non-descript baseline, occasionally interrupted by sharp transients (Figure 3). No focal, paroxysmal, or clinical features of seizure were observed. These EEG findings indicated severe diffuse electrocerebral impairment, with no discernible relationship between the observed movements and brain activity. Consequently, the movements were classified as myoclonic by the neurocritical care intensivist, which persisted despite treatment with valproic acid and levetiracetam. Given the persistence of the patient's myoclonus and severe encephalopathy, further investigation was warranted to elucidate the underlying etiology. Consequently, despite the lack of mental improvement observed during the sedation vacation, a CT scan of the head was performed before and after the administration of intravenous contrast on the fourth day of admission (Figure 4). The results revealed evidence suggestive of increased intracranial pressure and mild cerebral edema, characterized by the loss of the supratentorial grey-white matter interface. Subsequently, on the sixth day of admission, magnetic resonance imaging (MRI) of the brain without intravenous contrast was performed, which revealed diffuse diffusion restriction affecting the cerebral cortex, deep cerebral gray matter, and bilateral basal ganglia. Additionally, partial sulcal effacement and mild diffuse cerebral edema were also observed in the study. These findings were consistent with the sequelae of global metabolic brain injury, a pattern often associated with hypoglycemia (Figure 5).

The patient remained intubated throughout her hospital course and was maintained on minimal ventilatory settings. Despite supportive medical management, the patient's medical condition did not improve; therefore, the family was thoroughly briefed on the sequence of events and prognosis. Following an extensive discussion, a collective decision was reached to transition towards comfort-focused care with palliative extubation. The patient was pronounced dead 45 minutes after the palliative extubation.

## Discussion

The brain requires a constant and uninterrupted supply of glucose from the circulation to perform appropriate baseline functions, as it cannot synthesize glucose or store substantial amounts as glycogen in the astrocytes [6]. Animal studies have shown that neurological damage was associated with average blood glucose levels less than 0.7 mmol/L (13 mg/dL) [7]. The prevalence of hypoglycemia-induced irreversible brain injury is not known, as it is a rare complication seen after prolonged hypoglycemia for more than six hours [7-9].

Hypoglycemia-induced brain injury or hypoglycemic encephalopathy is often due to exogenous insulin use in diabetic patients, sepsis, alcoholism, drugs such as sulfonylureas, and hepatic, renal, and endocrine disorders [5,8]. An estimated 1.8% of patients receiving any form of insulin therapy develop severe hypoglycemia at least once annually [10]. Severe hypoglycemia is more commonly seen in patients treated with bolus insulin alone than in those treated with basal insulin alone [11]. In our case, the patient was on a basal bolus insulin regimen for her poorly controlled diabetes. As described above, our patient was never prescribed sulfonylureas, did not have any hepatic or renal dysfunction, and never showed any signs of sepsis.

Hypoglycemia causes active degradation of neurons. Various studies have discussed the pathogenesis of hypoglycemia-induced brain injury, which includes a combination of vascular disease, oxidative stress secondary to activation of neuronal nicotinamide adenine dinucleotide phosphate (NADPH) oxidase, neuroinflammation, mitochondrial dysfunction, as well as acetylcholinesterase (AChE) activation with accumulation of amyloid β and tau phosphorylation [12]. Rapid improvement of acute hypoglycemia can prevent the initiation of a cascade of neuronal cell death. Correction of serum glucose in prolonged hypoglycemia does not interrupt the process of neuronal death [6]. In our case, even though serum glucose was rapidly corrected on presentation, prolonged downtime of more than 12 hours led to progression of the neuronal cell death cascade.

Clinical signs of hypoglycemia are dependent on the duration and severity of the hypoglycemic event. Mild to moderate hypoglycemia for less than six hours presents as hunger, weakness, syncope, paroxysmal sweating, palpitations, and tremors. If symptoms persist for more than six hours, patients may feel lethargic, confused, delirious, and have impaired awareness. Severe hypoglycemia for more than six hours usually presents as an unresponsive patient with poor GCS scores [13,14]. Our patient presented with unconsciousness and a GCS score of 3, which did not improve following administration of dextrose, which signified that she had prolonged hypoglycemia of at least more than six hours. Patients with hypoglycemic encephalopathy may also have focal neurologic symptoms, including hemiplegia, hemianopia, aphasia, and convulsions [14]. In our case, a thorough workup was done to find the cause of the initial myoclonus and right-sided deviation of the eyes. Through extensive radiographic and electrical studies of the brain, underlying seizures, cerebrovascular accidents, and other organic or anatomic brain abnormalities were ruled out, and a source of myoclonus could not be identified. The patient further did not respond to anticonvulsants generally recommended for myoclonus, which included valproic acid and levetiracetam [15].

The Barbara et al. study done in 2017 showed that patients having hypoglycemia for more than eight hours suffered from poor neurological outcomes [16]. The study also revealed that care was more frequently withdrawn or withheld for patients suffering from prolonged hypoglycemia for an average of 12 hours. Studies have shown that the basal ganglia, substantia nigra, cerebral cortex and the hippocampus are more prone to acute injury in an event of acute severe hypoglycemia with sparing of the cerebellum, brain stem and thalamus [8]. This may be due to the higher activity of glucose transporters in the cerebellum and brainstem [17]. White matter involvement is seen in later stages of the disease. A unique finding that differentiates hypoglycemic brain injury from anoxic brain injury is the pattern of its neuropathologic distribution. Cortical lesions do not generally conform to a specific cerebro-arterial distribution in cases presenting with hypoglycemic brain injury. Hypoglycemia may also cause swelling of the capillary endothelial cells, causing circulatory disturbances and cerebral edema [16]. In some cases, although there may be an overlap of brain imaging findings where anoxic and hypoglycemic injuries may be compounded.

Our case conforms to the typical findings of severe, prolonged hypoglycemia-induced irreversible brain injury characterized by the diffuse involvement of the entire cerebral cortex and bilateral basal ganglia with mild diffuse cerebral edema. Despite a thorough workup, which included long-term EEG monitoring, CT, and MRI, no other source of the brain injury could be identified.

## Conclusions

Hypoglycemia should be promptly diagnosed and corrected to prevent life-threatening complications, including encephalopathy and death. Blood glucose levels are easily and accurately measured by a fingerstick blood glucose monitor. This case highlights the importance of patients on an insulin regimen to frequently monitor their glucose levels to prevent hypoglycemia. Patients must be educated thoroughly on the directions for using insulin and the importance of having meals following insulin administration.

Severe hypoglycemia with serum glucose levels of less than 20 mg/dL for a prolonged time of more than six hours leads to irreversible brain injury and neuronal death. Diagnosis of hypoglycemia-induced brain injury can be made based on clinical signs and symptoms. Radiographic imaging with MRI can be used to confirm the diagnosis. Diffusion-weighted imaging (DWI) using high b-values may also provide essential information to formulate a diagnosis. The routine use of continuous glucose monitors in high-risk patients could be used to prevent severe hypoglycemia.
